# A sialidosis type I cohort and a quantitative approach to multimodal ophthalmic imaging of the macular cherry-red spot

**DOI:** 10.1136/bjophthalmol-2020-316826

**Published:** 2020-07-31

**Authors:** Malena Daich Varela, Wadih M Zein, Camilo Toro, Catherine Groden, Jean Johnston, Laryssa A Huryn, Alessandra d’Azzo, Cynthia J Tifft, Edmond J FitzGibbon

**Affiliations:** 1 Ophthalmic Genetics and Visual Function Branch, National Eye Institute, Bethesda, Maryland, USA; 2 Medical Genetics Branch, National Human Genome Research Institute, Bethesda, Maryland, USA; 3 Department of Genetics, Saint Jude Children’s Research Hospital, Memphis, Tennessee, USA; 4 National Eye Institute, Bethesda, Maryland, USA

**Keywords:** Genetics, Macula, Imaging, Diagnostic tests/Investigation, Retina

## Abstract

**Aim:**

To describe the ophthalmologic findings on the largest cohort of patients with sialidosis type I due to deficiency of the lysosomal sialidase, neuraminidase 1 (NEU1) and to introduce a quantitative neuroretinal image analysis approach to the associated ‘macular cherry-red spot’.

**Methods:**

Seven patients with sialidosis type I (mutations in *NEU1*) and one with galactosialidosis (mutations in *CTSA*) were included. All patients underwent detailed ophthalmological examinations. The reflectivity of macular optical coherence tomography (OCT) was measured using greyscale analysis (Fiji) and compared with age-matched healthy volunteers. Four patients were evaluated over a time of 1.5+0.5 years.

**Results:**

The mean age of the patients at their first visit was 27.5+9.8 years. All patients had a macular cherry-red spot, clear corneas and visually non-significant lenticular opacities. The mean visual acuity was LogMar 0.4 (20/50)+0.4 (20/20 to 20/125). Six patients had good visual function. Optic atrophy was present in two individuals with reduced acuity. A significant increase in macular reflectivity was present in all patients compared to age-matched controls (p<0.0001).

**Conclusion:**

Most of our patients (75%) have preserved visual acuity, even in adulthood. The presence of optic atrophy is associated with poor visual acuity. Increased macular reflectivity by OCT greyscale measurements is noted in all patients, although the underlying biological basis is unknown. These findings complement the current methods for examining and monitoring disease progression, especially in patients for whom visualisation of the cherry-red spot is not entirely clear.

**Trial registration number:**

NCT00029965.

## INTRODUCTION

Sialidosis or mucolipidosis I (OMIM# 256550) and galactosialidosis (GS, OMIM**#** 256540) are two related disorders belonging to the glycoproteinosis subgroup of over 70 other lysosomal storage diseases.^1^ Sialidosis is a recessive disease resulting from mutations in the *NEU1* gene, which encodes neuraminidase 1 (NEU1). This enzyme hydrolyses the sialic acid residue of glycoproteins and glycolipids, as part of the lysosomal role of degradation of complex macromolecules.^2^ There are two defined clinical phenotypes.^3^ Sialidosis type I, often referred to as ‘myoclonus-cherry-red spot syndrome’, is a less severe disorder, usually presenting in the second decade of life with myoclonus, seizures, ataxia and visual decline. Sialidosis type II, a more severe, early-onset form of the disease (congenital or infantile) runs a more acute and often fulminant course.^4^

Galactosialidosis is a related recessive disorder caused by mutations in *CTSA* gene, which encodes the protective protein cathepsin A (PPCA).^5^ PPCA forms a stable tri-protein complex with NEU1 and beta-galactosidase (β-GAL). Primary PPCA deficiency leads to a secondary, combined deficiency of NEU1 and β-GAL. Galactosialidosis has three clinical phenotypes: early infantile, late infantile and juvenile/adult. The latter is the most common and may include coarse facies, skeletal anomalies, myoclonus, cherry-red macula and seizures.^5^

A macular cherry-red spot is a well-known feature of sialidosis, galactosialidosis and other lysosomal storage disorders such as infantile Tay-Sachs, Nieman-Pick and Sandhoff diseases.^6^ However, its relevance to patients’ visual acuity and its association with age, disease duration and prognosis for future visual function are uncertain. The purpose of this study is to provide a detailed description of the ophthalmologic findings in the largest cohort of patients with sialidosis type I to date. In addition, we report on one patient who initially presented as ‘myoclonus-cherry-red spot syndrome’ who was subsequently molecularly diagnosed with galactosialidosis. We expand on the characterisation of the prototypical features of the ‘cherry-red spot macula’ by providing comprehensive quantitative and multimodal imaging analyses.

## MATERIALS AND METHODS

Eight patients enrolled in an institutional review board-approved protocol; ‘Nervous System Degeneration in Glycosphingolipid Storage Disorders’ (ClinicalTrials.gov Identifier: NCT00029965) at the National Institutes of Health, Bethesda, Maryland, USA. At the time of enrollment, all eight were known to have elevated urinary sialyloligosaccharides, clinical evidence of a ‘cherry-red’ spot on fundus examination and neurological dysfunction including myoclonus, seizures and ataxia. Seven patients had biallelic mutations in the gene *NEU1*. The eighth, who was *NEU1* mutation-negative, was subsequently found to have biallelic mutations in *CTSA* confirming a diagnosis of galactosialidosis. Since the cell biological, biochemical and ophthalmological features of sialidosis and galactosialidosis overlap at many levels, we will refer to our cohort as a whole. Thus, 8 patients and 16 eyes were included in this study.

As part of a comprehensive natural history study, all patients underwent detailed evaluation by an ophthalmologist, who tailored the testing to the patients’ ability to participate in the exam. The exam included medical history, visual acuity measurement using ETDRS,^7^ Ishihara plates colour vision test, ocular motility and a slit lamp and fundus evaluation. Colour and red-free fundus photographs were acquired in the eight patients (Topcon Medical Systems, Oakland, New Jersey, USA). Four patients had fundus autofluorescence imaging (Topcon) and two had ultrawide-field imaging (Optos, Dunfermline, Scotland).

Optical coherence tomography (OCT) was obtained dilated, using Cirrus HD-OCT (Carl Zeiss Meditec, Dublin, California, USA). The scanning included retinal nerve fibre layer (RNFL) thickness and the macular cube protocol. The latter consists of the analysis of a 6 mm^2^ area of the macula by 128 horizontal lines, each consisting of 512 A-scans per line. Macular thickness was then calculated as a total and in nine different sections, according to the Age-Related Eye Disease Study subfields.^8^ The central subfield corresponds to a circular area of 1 mm diameter, centered around the fixation point and the inner subfields are limited to a concentrically larger ring measuring 3 mm in diameter, excluding the central subfield previously described.

Reflectivity of the OCTs was measured using greyscale analysis (Fiji/ImageJ2).^9^ We first selected the OCT cut that crossed through the foveola, from both the right and left eyes of each individual. Second, we drew a line in the middle of the foveola to divide the macular OCT into temporal and nasal subfields. Two line selections were then placed 700 μm nasal and temporal from the middle line and analysed using the Plot Profile tool. These lines started in the vitreous cavity and cross perpendicular to the retinal pigment epithelium (RPE) until reaching the choroid. We quantified the first peak value, which occurred when the line crossed from the vitreous cavity to the inner retina. To avoid false readings due to artefacts interfering with the overall OCT reflectivity, we normalised the data taking as the individual’s baseline the reflectivity of the entire region from the posterior border of the RNFL down to the posterior border of the RPE (standardisation used by Gardiner *et al*).^10^ Macular OCTs from 16 healthy volunteers were analysed for comparison. The normal subjects’ ages were ±5 years of one of the patients, they had mild to no refractive error and their exams were done dilated. We analysed both the macular structural parameters and the reflectivity of the inner layers of each of their eyes.

Statistical analysis of different variables was performed using GraphPad Prism 8.2.0, and the threshold to determine significance was p<0.05. One limitation of our statistical analyses, however, was the relatively small cohort. Therefore, we took into account all 16 eyes from affected individuals and 32 from healthy controls when performing the calculations.

A follow-up evaluation was obtained in four patients (50%). Genetic, biochemical and systemic information of these patients was provided by the primary team and these results were tabulated.

## RESULTS

A summary of the ocular findings in our cohort is described in table 1.

**Table 1:** Summary of ocular findings among our cohort

Number of patients	8
Mean age (years)	27.5
Visual disability	2/8; 25%
Lens opacities	8/8; 100%
Optic atrophy	2/8; 25%
Macular cherry-red spot	8/8; 100%
Color vision defects	0/5; 0%*

*Different denominator due to test not performed in all patients.

The mean age of our cohort at their first visit was 27.5±9.8 years, with a median of 29.5 years. Patients I and II were in the pediatric age group, aged 12 and 14 years old at their first visit, respectively. Four of our patients were female and four were male. Two of these eight patients were siblings (Patients III and VII) and the rest of the cohort did not have a family history of sialidosis, galactosialidosis or other lysosomal storage disease. Three patients were African American (II, V and VI) and the remaining five were Caucasian.

The mean visual acuity of our cohort was LogMar 0.4 (20/50) ±0.4 (20/20 to 20/125) in the right (OD) and left (OS) eyes, with a median of 20/40 OD and 20/32 OS. Six patients (75%) had good visual function (20/60 or better OD and OS), Patient VIII was visually impaired (20/100 OD and OS) and Patient IV was legally blind (20/250 OD and OS). There was no significant association between age and visual acuity (linear regression, p=0.37). Patients II, VI and VIII (37.5%) were moderate and high myopes, with spherical equivalents of −5.50 D, −6.50 D and −11.00 D, respectively. The remaining five had minor to no refractive error. Colour vision was tested in five of the eight patients and the result was normal in all of them.

On motility exam, five patients had normal ductions, pursuits and saccades. Among the remaining three, Patient V showed slow horizontal saccades, Patient VIII had saccadic pursuit and Patient IV presented with slow horizontal saccades and saccadic pursuit, as well as occasional slow-amplitude and large-amplitude vertical pendular nystagmus.

**Figure 1:**
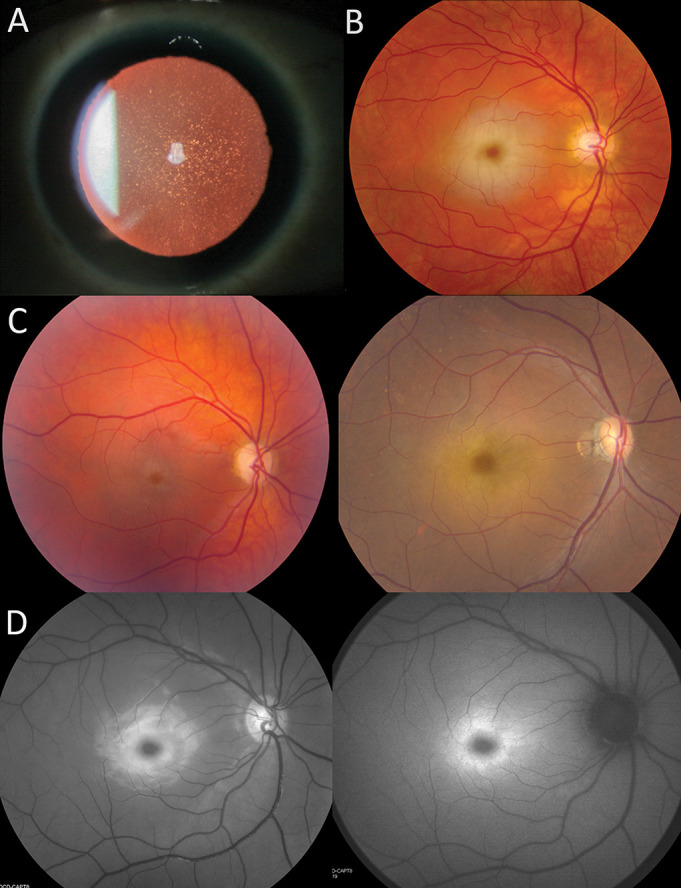
Ophthalmological features of patients with sialidosis type I. (A) Cortical and nuclear dot-like lens opacities (Patient VI). (B) Colour retinography showing a classic macular cherry-red spot (Patient VII). (C) Colour retinography showing a faded macular cherry-red spot in Patients IV (left) and VIII (right). (D) Multimodal imaging showing an infrared retinography on the left and autofluorescence imaging on the right (both from Patient I).

The corneas of our patients were clear. All of them (100%) presented visually non-significant changes on their lenses (figure 1A), such as scattered white speckles and punctate opacities affecting the cortex or the nucleus.

The two patients who had decreased visual acuity had pale optic discs and the thinnest RNFL of the cohort: 68 μm in Patient IV and 72 μm in Patient VIII. The other six patients had normal optic disc colour and cupping. The remaining four adults had a mean RNFL thickness of 121.2±10 μm, whereas the two children (Patients I and II) had a mean RNFL of 93 μm. There was a statistically significant correlation between RNFL thickness OD and OS (Spearman correlation, p<0.001, R=0.93) and no significant differences with the group of healthy volunteers (unpaired t-test, p=0.97). RNFL thickness was not associated with patients’ age (linear regression, p=0.81), but it showed a statistically significant association with visual acuity (linear regression, p=0.001).

All patients presented with a macular cherry-red spot (figure 1B). African American patients presented with a darker ‘cherry-brown’ spot due to the increased pigment density in their RPE. Qualitatively, the patient with the least clear macular cherry-red spot by colour fundus imaging was Patient IV, followed by Patient VIII (figure 1C). This difficulty was due to a faded pale ring surrounding the obscured fovea, causing a less striking contrast between the foveal spot and the retina. The remaining six patients had a prototypical, obvious macular cherry-red spot. Both in red-free and in autofluorescence imaging, the pale ring surrounding the obscured fovea is enhanced, making it easier to discern the presence of the macular cherry-red spot (figure 1D).

While analysing the macular OCT studies, we took into consideration structural parameters and reflectivity. The mean macular volume of our cohort was 9.95±1.02 mm^3^, the mean central foveal thickness (1 mm around the fixation point) was 263.5±22 μm and the mean thickness of the midperipheral ring area (3 mm area around the fixation point) was 325±33 μm, averaging the superior, inferior, nasal and temporal subfields. There was a significant correlation of these three macular parameters between OD and OS (Spearman correlation, p=0.0004, R=0.89, p=0.005, R=0.76, p<0.0001, R=0.95, respectively) and no significant differences between these values and those of healthy volunteers. There were no significant associations between these structural values and age (linear regression, p=0.3, p=0.13 and p=0.91, respectively) or visual acuity. As mentioned by Yamazaki *et al*, due to the increased reflectivity of the ganglion cell layer (GCL) in sialidosis patients, the limit between this and the RNFL is difficult to determine and therefore quantifying the thickness of GCL becomes unreliable.^11 12^ In our case, both automatic and manual measurements were attempted and disregarded due to high variability and poor correlation with the other structural parameters.

**Figure 2:**
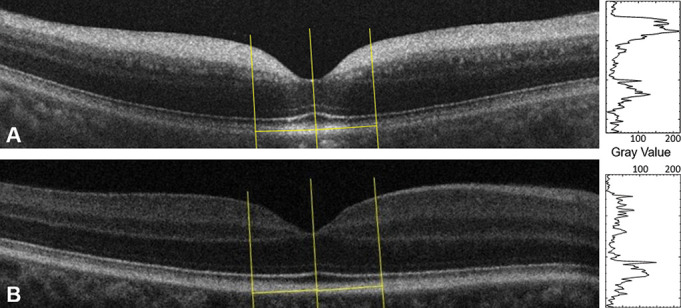
Macular optical coherence tomography (OCT) showing the line selections traced with Fiji to measure reflectivity. (A) Patient V (OD), (B) 30-year-old healthy volunteer (OD). The greyscale values of each macular OCT are graphed to the right of the image.

Another remarkable characteristic of these patients’ macular OCTs was the high reflectivity of the inner layers. The highest peak in our cohort was found in the inner retinal layers, while for the healthy volunteers, this was localised in the outer retina or RPE (figure 2). The mean reflectivity of the inner layers on the patients’ OCTs was 313±72, and on the normal subjects, 135.4±10.4. We found a statistically significant association between the greyscale values of the nasal and temporal lines (linear regression, p<0.0001), and a significant correlation between OD and OS both in patients (Spearman correlation p=0.0004, R=0.98) and in normal subjects (p=0.0009, R=0.76). We then compared the measurements from both of our patients’ eyes (figure 2A) with the ones of healthy volunteers (figure 2B) and found a statistically significant difference (unpaired t-test, p<0.0001). There was a statistically significant association between the greyscale values from our patients and their RNFL thickness (linear regression, p=0.006), and not significant with macular volume (linear regression, p=0.45), foveal thickness (linear regression, p=0.67) and midperipheral macular thickness (linear regression, p=0.67). We also compared the greyscale values with age and did not find an association in our patients (linear regression, p=0.09) nor in the healthy volunteers (linear regression, p=0.88). Greyscale was not associated with visual acuity either (linear regression, p=0.29). The macular reflectivity values from our cohort and the normal subjects are represented in table 2.

**Table 2:** Reflectivity of OCT’s inner layers from patients and age-matched healthy volunteers (HV)

Patient	Macular OCT reflectivity OD (greyscale)	Macular OCT reflectivity OS (greyscale)	Macular OCT reflectivity of age matched HVs OD (greyscale)	Macular OCT reflectivity of age matched HVs OS (greyscale)
1	220%	218%	143%	152%
			152%	143%
2	276%	264%	139%	137%
			123%	122%
3	288%	306%	125%	129%
			121.50%	132%
4	321%	273%	121%	126%
			123%	132%
5	359%	314%	145%	136%
			134.50%	136%
6	384%	377%	144%	148%
			122%	126%
7	445%	451%	153%	147%
			151%	138%
8	267%	245%	133%	123%
			144%	133%

Data was normalised taking as the individual’s baseline the reflectivity of the complete region from the posterior border of the retinal nerve fibre layer down to the posterior border of the retinal pigment epithelium.

HV, healthy volunteer; OCT, optical coherence tomography.

### Follow-up

Four patients were evaluated over a mean follow-up time of 1.5±0.5 years. All of them showed stable visual acuity and fundus evaluation. OCT structural parameters and reflectivity were also unchanged or with mild, non-significant variability.

### Genetics, Biochemical and Systemic data

All patients had comprehensive clinical, molecular and biochemical investigations. Some of the molecular and biochemical information is described as part of a larger cohort study by Mosca *et al* ^13^ and is the subject of an additional sialidosis type I natural history study underway (Tifft and Toro in preparation).

A detailed summary of the ophthalmological characteristics of our cohort is described in table 3.

**Table 3:** Demographic and ocular characteristics of our cohort at their most recent evaluation

Patient	Age (years)	BCVA OD	BCVA OS	Macular volume OD (mm^3^)	Macular volume OS (mm^3)^	CFT OD (μm)	CFT OS (μm)	Middle ring thickness OD (μm)	Middle ring thickness OS (μm)	RNFL thickness OD (μm)	RNFL thickness OS (μm)
1	12	20/32	20/25	10.2	10.4	267	272	345.25	345.25	98	82
2	16	20/40	20/32	10	10	272	266	335.75	333.5	96	95
3	29	20/20	20/20	11	11.2	309	314	373.5	376.75	110	116
4	30	20/250	20/250	8.9	8.7	255	243	266.25	260.5	67	69
5	31	20/30	20/30	11.2	11.1	267	269	348.25	342.25	132	122
6	33	20/20	20/25	10	8.5	248	244	317.5	310.75	105	116
7	40	20/20	20/20	10.6	10.6	283	274	342	341.25	117	129
8	41	20/100	20/200	8.8	8.8	233	235	287.75	288	71	74

BCVA, best corrected visual acuity; CFT, central foveal thickness; OD, right eye, OS, left eye; RNFL, retinal nerve fibre layer.

## DISCUSSION

The ophthalmological spectrum of sialidosis type I is characterised by a progressive decline in visual acuity, macular cherry-red spot and diffuse mild lens opacities.^14^ The basis for the progressive vision loss has not been clearly established, but it has been speculated that it is driven by progressive optic atrophy,^7 15^ yet, review of the literature offers a somewhat conflicting account.^4 16–27^ As lysosomal storage disorders, both sialidosis and galactosialidosis are associated with progressive lysosomal accumulation of undigested sialylated substrates, especially in neural tissue. This process drives neuronal cell death and neurodegenerative symptoms progression. A macular cherry-red spot in other lysosomal storage diseases, including sialidosis and galactosialidosis, is caused by substrate accumulation within the retinal GCL.^6^ Although histopathology studies have not been reported for sialidosis and galactosialidosis, this was clearly described for Tay-Sachs disease.^28^

On our literature review of 17 reported sialidosis cases including ophthalmological data, a macular cherry-red spot was often a key finding prompting further metabolic and molecular investigations which ultimately lead to an accurate molecular diagnosis.^4 16–27^ As to other ophthalmological features, the data is conflicting. Decreased visual acuity was present in five cases (29%), none of which were reported to have optic atrophy.^4 21 22 24 26^ Three patients (18%) were found to have optic atrophy, all with good visual function (ages 14, 15 and 21).^18 25^ Lowden and O’Brien reviewed the cases of sialidosis published before 1979 and found decreased visual acuity in all cases (n=10).^29^ Caciotti *et al* described a cohort of five patients, only two of which had decreased visual acuity (ages 13 and 19), but none had reported optic atrophy.^30^ In aggregate, these studies would suggest that the presence of a cherry-red spot offers relatively little additional information on rate of disease progression or long-term visual function. These ambiguities and inconsistencies reinforce the need for longitudinal observational studies such as ours. Our cohort is the largest to date with comprehensive multimodal ophthalmic investigation in patients with sialidosis type I, covering a wide age range (from 12 to 41 years old). Unlike the scattered case reports in the literature, having evaluated these patients at the same centre, with the same instruments and by the same team, makes these data suitable to be analysed as a group and provides a solid starting point for longitudinal exploration of visual function in this disease.

Regarding structural parameters, the mean macular volume of our cohort (9.95±1.02 mm^3^) was mildly below what was reported on a healthy population (10.01±0.02 mm^3^).^31^ The mean central foveal thickness (263.5±22 μm), however, was moderately above the normal limits (255.4±0.9 μm).^31^ Five patients were above this limit on both eyes and the remaining three (Patients IV, VI and VIII) were below on at least one eye. Similarly, the mean thickness of the midperipheral ring area (325±33 μm) was moderately greater than the one described in the normal population (316.5±0.6 μm),^31^ Patients IV, VI and VIII had values below the normal limit on at least one eye and the remaining five had appropriate thickness. Patients IV and VIII were also the only ones presenting with decreased RNFL thickness, below the normally reported among adults (99.4±9.7 μm).^32^ Interestingly, the two children (Patients I and II) had a mean RNFL (93 μm) that corresponds to the 5th percentile of their age group (normal RNFL thickness in children: 109±15.5 μm).^33^ We did not find an association between age and any parameter on our patients’ evaluation (visual acuity, RNFL thickness, macular volume, macular thickness, reflectivity of the inner retinal layers). Likewise, all patients presented with clear corneas and non-visually significant changes in the lens, even at an older age. Therefore, we do not find that age itself is responsible for their acuity loss.

The OCT reflectivity pattern seen in individuals with sialidosis has been described on case reports in the past: hyper-reflective inner layers adjacent to the macula, relative hypo-reflectivity of the layers underneath and apparent hyper-reflectivity of the photoreceptor layer in the foveal region.^19 25 26^ Ours would be the first attempt to quantify this characteristic on these individuals, providing an additional tool for when the diagnosis is not clear.

Quantifying OCT’s reflectivity has been used for different purposes over the past years, such as analysing cystic spaces in cystoid macular oedema, evaluating individuals with glaucoma and studying reticular pseudodrusen.^10 34–38^ Different software has been used to assess this parameter by detecting pixels’ reflectivity^35 37^ or measuring tissue’s retardance at different retinal locations.^39^ Several factors can interfere with the overall reflectance of OCT, such as pupil dilation, variations on the focal plane or point of the incident light, media opacity and corneal aberrations, among others. Thus, standardising the values allows us to better depict the particularities of the layer we are aiming to analyse, avoiding false readings due to imaging artefacts. Farci *et al* normalised the greyscale by subtracting the value inside the macular cysts from the one outside of them, Paavo *et al* do not mention standardisation of their data and Vermeer *et al* used RPE reflectivity as a reference layer.^34 35 38^ Gardiner *et al* studied RNFL’s reflectivity on glaucoma patients and compared different tissues as reference to standardise their data: posterior vitreous, RPE and the complete region from the posterior border of the RNFL down to the posterior border of the RPE.^10^ They found that the coefficients of variation were minimised by using the latter as the reference layer. Therefore, we used the same region to normalise our results and found that the variability among controls decreased indeed and the values better represented the particular reflectivity of the tissues. A strength from our paper is that the previous ones that evaluated RNFL used custom software, while Fiji is an open-source platform, allowing a more accessible analysis.^10 35–37 39^

Unlike other causes of sporadic macular cherry-red spot (such as central retinal artery occlusion) where the thickening of the macula is often present,^40^ we found patients with macular thickness values above and below the expected range and, as a group, there was no statistically significant difference between our patients and the healthy volunteers. The only significant difference between our patients and volunteers’ macular OCTs was the calculated greyscale, an indicator of abnormal reflectivity of the retinal inner layers. In our patients, these values were significantly associated with RNFL thickness only. Hence, analysing the macular greyscale could be a useful tool in cases where quantitative measurements of the RNFL are not reliable or to complement them as part of the patient’s evaluation, possibly detecting subtle changes.

High reflectivity was also noticed in patients with macular cherry-red spot secondary to central retinal artery occlusion.^40^ Although the pathophysiology is different, in both cases, the abnormal reflectivity may result from intracellular accumulation of liquid (oedema) or lipids within the retinal GCL.^28^ This becomes more noticeable in the perifovea due to the higher density of these cells in that area when compared to the rest of the retina and contrasts with the lack of inner retinal layers in the fovea, creating the cherry-red spot effect.^41^ By OCT, this intracellular accumulation results in higher reflectivity. The histopathological basis for these findings in sialidosis and galactosialidosis is unknown, but if they indeed relate to undigested lysosomal substrate accumulation, inner retinal reflectivity could be a sensitive and useful tool to monitor substrate burden within the retina, possibly detecting changes earlier than other structural parameters.

There are several cases of sialidosis type I in the literature where the cherry-red spot was not clear resulting in a delayed diagnosis.^4 21^ Bou Ghannam *et al* reported a patient without macular cherry-red spot but with thickened OCT and hyperautofluorescence perifoveally.^42^ Analysing only macular thickness can lead us to false negatives due to this parameter being altered by the patients’ refraction. Evaluating the greyscale of the macular OCT can be particularly useful when the prototypical sign is not clinically evident.

Since our observations include only cross-sectional data, short follow-up interval and patients with a heterogeneous combination of pathogenic alleles on a diverse genetic background, we cannot rule out the possibility that longitudinal natural history of the retinal thickness could follow a ‘biphasic’ pattern with thickening and increased reflectivity early in the disease followed, over time, by progressive retinal GCL degeneration with reduced greyscale values, eventual retinal thinning, optic atrophy and decreased visual acuity as found in our most visually affected cases. This line of inquiry can be formally explored on existing animal models of sialidosis such as the one described by d’Azzo *et al* and Bonten *et al.* ^3,43^

When considering ophthalmological findings in sialidosis as possible outcome measures for future disease-modifying interventional clinical trials, we believe monitoring structural OCT findings as well as macular greyscale parameters to be valuable. Most of our patients presented with good visual acuity, and age was not associated with decreased vision. Quantifying macular OCTs’ reflectivity can be particularly useful in patients with lysosomal storage disorders, especially in cases where the cherry-red spot is not clear.
